# Elderberry (*Sambucus nigra* L.) Fruit Extract Alleviates Oxidative Stress, Insulin Resistance, and Inflammation in Hypertrophied 3T3-L1 Adipocytes and Activated RAW 264.7 Macrophages

**DOI:** 10.3390/foods8080326

**Published:** 2019-08-08

**Authors:** Joanna Zielińska-Wasielica, Anna Olejnik, Katarzyna Kowalska, Mariola Olkowicz, Radosław Dembczyński

**Affiliations:** 1 Department of Biotechnology and Food Microbiology, Poznan University of Life Sciences, Wojska Polskiego 48, 60-627 Poznan, Poland; 2 Department of Chemistry, University of Waterloo, 200 University Avenue West, Waterloo, ON N2L 3G1, Canada

**Keywords:** elderberry polyphenols, functional food, obesity, digestive enzymes, fat cells, intracellular reactive oxygen species, adipokines, glucose uptake, immune-metabolic effects

## Abstract

Oxidative stress and inflammation in hypertrophied adipose tissue with excessive fat accumulation play a crucial role in the development of obesity and accompanying metabolic dysfunctions. This study demonstrated the capacity of elderberry fruit (EDB) extract to decrease the elevated production of reactive oxygen species in hypertrophied 3T3-L1 adipocytes. Treatment with the EDB extract resulted in modulation of mRNA expression and protein secretion of key adipokines in hypertrophied adipocytes. Expression of leptin and adiponectin was, respectively, down- and up-regulated. Moreover, glucose uptake stimulation was noticed in mature adipocytes, both sensitive to insulin and insulin resistant. This may suggest a positive effect of EDB extract on insulin resistance status. The extract was also found to alleviate the inflammatory response in activated RAW 264.7 macrophages by down-regulating the expression of proinflammatory genes (*TNF-α*, *IL-6*, *COX-2*, *iNOS*) and suppressing the enhanced production of inflammatory mediators (TNF-α, IL-6, PGE_2_, NO). *In vitro* experiments showed that the EDB extract could inhibit digestive enzymes, including *α*-amylase, *α*-glucosidase, and pancreatic lipase, leading to reduced intestinal absorption of dietary lipids and carbohydrates. Further *in vivo* studies could be postulated to support EDB as a functional food component for the prevention and treatment of obesity and metabolic-immune comorbidities.

## 1. Introduction

Obesity is associated with excessive adipose tissue growth, which occurs through two possible mechanisms: hypertrophy (expansion of existing adipocytes) and hyperplasia (recruitment of new adipocytes). Hypertrophic adipose tissue growth is mainly considered to be related to insulin resistance and other obesity metabolic comorbidities [1]. Abnormal expansion of adipose tissue is accompanied by local hypoxia, adipocyte death, enhanced cytokine and chemokine secretion, dysfunctional fatty acid metabolism and accumulation, and immune cell infiltration. Dysregulation of lipid metabolism in adipose tissue leads to enhanced release of free fatty acids, which initiates inflammatory signaling cascades in the infiltrating cell population. Chronic low-grade inflammation, found in abnormal fat tissue, negatively affects the insulin signal transduction pathway, and promotes insulin resistance [2,3].

Recent scientific preclinical studies have shown that bioactive dietary compounds may specifically influence hypertrophic adipose cells and mitigate the effects of extensive adipose tissue growth by affecting various adverse phenomena, including oxidative stress, inflammation, disturbances in adipokine secretion, fatty acid release, and others. Berry fruits have been recognized as capable of counteracting obesity and obesity-related metabolic disorders, through the inhibition of adipocyte differentiation, a decrease in lipogenesis, an increase in lipolysis, or mitigation of inflammatory and insulin resistance status [4].

A promising candidate capable of attenuating obesity and complications related to excessive fat tissue growth might be *Sambucus nigra* L. (European elderberry) fruit as a valuable source of polyphenolic compounds, primarily flavonols, flavanols, phenolic acids, proanthocyanidins, and anthocyanins [5]. The unique polyphenol composition is responsible for the high biological potential of elderberry fruit (EDB), including antiviral and antimicrobial activity, as well as chemopreventive, neuroprotective, and anti-inflammatory effects that have been documented in several scientific reports [6,7,8,9,10]. Also, it has been suggested that EDB may be an effective remedy for diabetes, obesity, and metabolic dysfunctions [9]. Animal studies have shown the ability of *Sambucus nigra* preparations to improve glucose and lipid metabolism and diabetic osteoporosis status [11,12,13,14].

Anthocyanin-rich EDB extract has been proved to attenuate systemic inflammation and insulin resistance in high-fat diet-induced obese mice. Pro-inflammatory markers of low-grade chronic inflammation, including serum monocyte chemoattractant protein-1 (MCP-1) and tumor necrosis factor-α (TNF-α), were significantly reduced in EDB-fed mice. Also, the high-fat diet supplemented with EDB extract mitigated some metabolic disturbances by lowering serum triglycerides and improving insulin sensitivity [12]. Lowered insulin resistance was found in diabetic rats fed with a high-fat diet supplemented with EDB extracts rich in triterpenic acids or polyphenol compounds. The extracts modulated glucose metabolism by correcting hyperglycemia or reducing insulin secretion, respectively [13]. The anthocyanin-rich EDB extract protected against inflammation-related impairments in high-density lipoprotein (HDL) function in a mouse model of hyperlipidemia and HDL dysfunction. The decrease in total cholesterol content of the aorta in EDB-fed mice suggested limiting atherosclerosis progression [14]. Scientific reports indicate that EDB extracts possess the unique potential to modulate the immune response depending on the immune stimuli and inflammatory disorders. The EDB bioactives have evoked different immune effects by controlling pro- and anti-inflammatory cytokines and mediators (Reactive oxygen species, NO, IL-6, TNF-α, MCP-1, IL-1, IL-8, IL-10, PGE_2_, COX-2, iNOS, INF-γ), that play a crucial role in acute and chronic low-grade inflammatory diseases associated with obesity, diabetes, dyslipidemia, cardiovascular disturbances, and neurodegenerative diseases [7,8,10,11,12,13,14,15,16].

Over the last decade, significant advances in knowledge about the health-beneficial potential of EDB fruit have been achieved through extensive preclinical studies. However, the results obtained only in the few clinical trials have not enabled to express an unambiguous opinion and, so far, have not provided strong evidence of the therapeutic effects of *Sambucus nigra* fruit in obesity and metabolic disorders [9]. Recently, the scientific community has stated the need for further research on the health-promoting properties of this valuable plant as a natural constituent of food products and beneficial component of a healthy diet [6,9].

This study aimed to evaluate the capacity of *Sambucus nigra* fruit extract to mitigate obesity-related metabolic complications through the carbohydrate and lipid metabolism regulation, glucose uptake improvement, and insulin sensitivity controlling. Also, the goal of the study was the assessment of the ability of the extract to alleviate the inflammatory response in activated macrophages, which are recruited into excessively growing fat tissue and may be a primary source of locally produced pro-inflammatory mediators.

## 2. Materials and Methods 

### 2.1. Preparation of Elderberry Fruit Extract

The fruits of elderberry (*Sambucus nigra* L.) cultivar Sampo, obtained from Bio Berry Poland (Warsaw, Poland), were homogenized to fruit pulp, which was subsequently frozen at −80 °C and subjected to freeze-drying at a vacuum pressure of 0.1 mbar and temperature of 20 °C for 23 h and post-drying at 23 °C for 3 h using a freeze dryer (LMC-1, Martin Christ Gefriertrocknungsanlagen GmbH, Germany). The lyophilized EDB were finely ground and packaged under nitrogen atmosphere. The EDB extract was obtained by dissolving the EDB powder in complete culture medium with the pH adjustment to 7.4. The EDB suspension was then centrifuged (3000 g, 5 min) and filtered through a 0.22 µm membrane (Merck, Germany).

### 2.2. Determination of Individual Phenolic Compounds Using HPLC-DAD-MS^n^ Analysis

Analyses of phenolic compounds were performed on an Agilent 1200 series HPLC system (Agilent Technologies, Inc., Santa Clara, CA, USA) that was equipped with a G1315D photodiode array detector and coupled online with an Agilent 6224 time-of-flight MS system. Phenolic compounds were identified using a mass spectrometer fitted with an electrospray ionization (ESI) source that was operated in positive-ion or negative-ion mode. Analyses were carried out using full MS scan mode, and full mass spectra were recorded in the range of 100 to 1700 *m*/*z*. Technical specification of apparatus and major HPLC/MS parameters and analysis conditions were described in detail in our previous work [17].

For quantification purposes, all anthocyanins conjugates were expressed as cyanidin-3-glucoside equivalents; all flavan-3-ols and their polymers as catechin equivalents; hydroxybenzoic acid glucoside and hydrolysable tannins as gallic acid equivalents; phenolic acids derivatives as chlorogenic acid equivalents; and flavonol glycosides as quercetin equivalents.

### 2.3. T3-L1 Cell Culture, Differentiation, and Treatment 

The mouse embryo 3T3-L1 cell line was purchased from the American Type Culture Collection (ATCC, CL-173). The 3T3-L1 preadipocytes were grown, passaged, and differentiated into adipocytes as described previously [18]. The 3T3-L1 cells were grown in Dulbecco’s Modified Eagle’s Medium (DMEM) with 10% calf serum supplementation (Sigma-Aldrich, Merck Group, Darmstadt, Germany). Cell differentiation was induced in post-confluent cell cultures by a differentiation mixture consisting of 1 μM insulin, 0.25 μM dexamethasone (DEX), and 0.5 mM 3-isobutyl-1-methylxanthine (IBMX) in DMEM with 10% fetal bovine serum (FBS) (Gibco, Thermo Fisher Scientific Polska, Warsaw, Poland). 

Fully differentiated 3T3-L1 cells were exposed to the EDB extract at concentrations of 5, 10, and 20 mg/mL for 24 h. The levels of intracellular ROS generation and lipid accumulation in mature adipocytes were determined. Also, the viability and metabolic activity of the mature adipocytes were analyzed after the treatment. 

After completion of the differentiation process, insulin resistance was induced in 3T3-L1 adipocytes by 10 ng/mL murine TNF-α (Sigma-Aldrich) for 5 days, with medium/TNF-α replacement every 2 days. Glucose uptake measurement was performed in insulin-resistant and insulin-sensitive adipocytes subjected to the EDB treatment. 

### 2.4. Macrophage Cell Culture and Anti-Inflammatory Experiment Procedure

RAW 264.7 murine macrophage line was obtained from the European Collection of Authenticated Cell Cultures (ECACC, 91062702) and supplied by Sigma-Aldrich. Cells were grown in DMEM supplemented with 10% heat-inactivated FBS at 37 °C in a humidified, 5% CO_2_, 95% air atmosphere. The 24-h cultures of RAW 264.7 macrophages, seeded at a density of 5 × 10^5^ cells/cm^2^, were treated with EDB extract prepared in DMEM at the concentrations of 0.1, 1, and 10 µg/mL and incubated for 2 h in standard culture conditions. Controls were treated with DMEM only. Subsequently, macrophages were stimulated with 5 ng/mL of lipopolysaccharide (LPS) from *Escherichia coli* O-127 (Sigma-Aldrich). After 3-h macrophage activation, the culture media and cells were harvested to analyze the protein secretion and gene expression of pro-inflammatory mediators.

### 2.5. Cell Viability Assay

The viability and metabolic activity of differentiated 3T3-L1 adipocytes and LPS-stimulated RAW 264.7 macrophages were analyzed using the MTT (3-(4,5-dimethylthiazol-2-yl)-2,5-diphenyltetrazolium bromide) test (Sigma–Aldrich) following the protocol described previously [8].

### 2.6. Measurement of Reactive Oxygen Species in Adipocytes

The intracellular ROS generation was determined using nitro blue tetrazolium (NBT) according to the procedure described by Choi et al. [19]. The cells were incubated in 0.2% NBT solution for 90 min, washed with phosphate-buffered saline (PBS), fixed with methanol, and then air-dried. The formazan extraction was performed using KOH and DMSO for dissolving. The absorbance was measured at 620 nm using a Tecan M200 Infinite microplate reader (Tecan Group Ltd., Männedorf, Switzerland).

### 2.7. Measurement of Intracellular Triglyceride Content in Adipocytes

Total concentrations of triglycerides (TG) in differentiated 3T3-L1 adipocytes were determined using Adipogenesis Assay Kit (Sigma-Aldrich) according to the manufacturer’s protocol. Intracellular TG content was measured by a coupled enzyme assay, which resulted in a fluorometric product detected at λ_ex_ = 535 nm and λ_em_ = 587 nm (Tecan M200 Infinite), which was proportional to the TG present. The TG concentration was calculated based on the curve plotted for TG standards. 

### 2.8. Glucose Uptake Measurement in Adipocytes

Glucose uptake assay was performed according to the modified method of Alonso-Castro and Salazar-Olivo [20]. Mature 3T3-L1 adipocytes, cultured on 24-well plates for fluorescence-based assays, were starved in serum-free medium (MEM containing BSA 0.5%) overnight. Subsequently, the medium was replaced with Krebs Ringer phosphate HEPES (KRPH) buffer containing 0.2% BSA (KRPH/BSA) and incubated for 60 min. The cells were then exposed for 60 min to EDB extract suspended in KRPH/BSA buffer supplemented with 80 μM 2-NBDG (2-*N*-7-(nitrobenz-2-oxa-1,3-diazol-4-yl) amino-2-deoxy-d-glucose) (Sigma-Aldrich) used as fluorescent glucose analogue. The control cultures were treated with 100 nM insulin or 10 μM rosiglitazone (Sigma-Aldrich). After incubation, cultures were immediately washed three times with ice-cold PBS. The fluorescence intensity of 2-NBDG was measured at λ_ex_ = 485 nm and λ_em_ = 535 nm (Tecan M200 Infinite). 

### 2.9. Determination of Adipokine Production in 3T3-L1 Adipocytes

The leptin and adiponectin concentrations were measured using ELISA kits (Sigma-Aldrich, Merck Group) following the manufacturer’s instructions. The adipokine concentrations were expressed in ng/mL of culture medium, which was equivalent to the amount of protein per 1 × 10^6^ cells.

### 2.10. Determination of IL-6, TNF-α, and PGE_2_ Production in RAW 264.7 Macrophages

The secretion of IL-6 and TNF-α cytokines as well as generation of PGE_2_ by LPS-stimulated RAW 264.7 macrophages were determined with ELISA kits (R&D Systems, Inc, Minneapolis, MN, USA) according to the manufacturer’s instructions. Protein concentrations were expressed in pg/mL of culture supernatant, which was equivalent to the amount of protein per 1 × 10^6^ cells.

### 2.11. Determination of NO Production in RAW 264.7 Macrophages

Griess method was applied to determine nitrite as an indicator of NO production. Equal volumes of the Griess reagent (Sigma-Aldrich) and RAW 264.7 culture supernatant were mixed and incubated at room temperature for 15 min. The absorbance was measured at 540 nm (Tecan M200 Infinite). The standard curve plotted for sodium nitrite was used to calculate NO concentration.

### 2.12. Quantification of Gene Expression Using Real-Time PCR

The analysis of gene expression was carried out in accordance with the detailed protocol presented in the previous work [17]. The TRI reagent (Sigma-Aldrich) was used to isolate total RNA, Synthesis cDNA Transcriptor First-Strand kit (Roche Diagnostics GmbH, Mannheim, Germany) for first-strand cDNA synthesis, and SYBR1 Select Master Mix (Life Technologies, Carlsbad, CA, USA) for real-time PCR. The primers used for the amplification of cDNAs are listed in Table 1.

The relative expression of each gene was calculated using the 2^−ΔΔCT^ method. The mRNA levels in the control cells were designated as 1, and the relative levels of the gene transcripts in the samples were expressed as the fold change.

### 2.13. Digestive Enzyme Inhibition Assays

#### 2.13.1. Measurement of Pancreatic Lipase Inhibition 

The EDB inhibitory activity against pancreatic lipase (EC 3.1.1.3) was evaluated according to the method of Boath et al. with minor modification [21]. The p-nitrophenyl laurate (pNP laurate) was used as a substrate. The pNP laurate was dissolved to 0.08% in 5 mM sodium acetate (pH 5.2) containing 1% Triton X-100 and 0.05% Arabic gum. The reaction mixture consisting of 350 μL of assay buffer (100 mM Tris, pH 8.2), 50 μL of EDB extract, 150 μL of pancreatic lipase type II from porcine pancreas (10 mg/mL), and 450 μL of substrate solution was incubated at 37 °C for 2 h. Orlistat, a known porcine pancreatic lipase inhibitor, was applied as a positive control. After incubation, the sample was centrifuged at 13,000 rpm for 3 min and read at 400 nm of wavelength (Tecan M200). 

#### 2.13.2. Measurement of α-Amylase Inhibition 

The inhibition of α-amylase (EC 3.2.1.1) activity was determined using the method of Tan et al. with slight modification [22]. The reaction mixture consisting of 200 μL of distilled water, 50 μL of EDB extract, 250 μL of α-amylase from porcine pancreas (30 mg/mL), and 500 μL of 0.5% starch was incubated at 37 °C for 10 min. Acarbose, a known pancreatic α-amylase inhibitor, was applied as a positive control. Enzymatically released reducing sugars were determined by DNS reagent solution (96 mM 3,5-dinitrosalicylic acid, 5.31 M sodium potassium tartrate in 2 M NaOH) after heating at 95 °C for 10 min. Then, the mixture was diluted with distilled water and the absorbance was measured at 540 nm (Tecan M200 Infinite). 

#### 2.13.3. Measurement of α-Glucosidase Inhibition 

The inhibition assay of α-glucosidase (EC 3.2.1.20) was adopted from Tan et al. [22]. The p-nitrophenyl-α-d-glucuronide (pNPG) dissolved to 4 mM in 0.1 M HEPES (pH 6.8) was used as a substrate. The reaction mixture consisting of 350 μL of HEPES (pH 6.8), 50 μL of EDB extract, 150 μL of α-glucosidase (20 mg/mL), and 450 μL of substrate solution was incubated at 37 °C for 2 h. The release of p-nitrophenol from the pNPG substrate was measured at 410 nm (Tecan M200 Infinite). As a positive control, the glucosidase inhibitor, acarbose, was used. 

All reagents used in digestive enzyme inhibition assays were provided by Sigma-Aldrich.

#### 2.13.4. Data Analysis

Enzyme activity in the presence of inhibitor (EDB extract or reference inhibitor) was expressed as a percentage of the non-inhibited enzyme activity and plotted versus inhibitor concentration. Based on the dose-response curve, the inhibitor concentration required for 10% and 50% inhibition of enzyme activity (IC_10_ and IC_50_) was determined as a measure of inhibitory potency. The percentage of the non-inhibited enzyme activity was calculated by following equation:

% non-inhibited enzyme activity = [(A_Inhibitor_ − A_Inhibitor blank_)/(A_Control_ − A_Control blank_)] × 100%

where A_Control_ is the absorbance of the sample without EDB extract/reference inhibitor; A_Inhibitor_ is the absorbance of the sample containing EDB extract/reference inhibitor; A_Inhibitor blank_ is the absorbance of the sample with EDB extract/reference inhibitor, but without enzyme addition; A_Control blank_ is the absorbance of the sample without EDB extract/reference inhibitor and enzyme addition. 

### 2.14. Statistical Analysis

All data are expressed as the means ± SD from three independent experiments. Statistical analysis was performed using the STATISTICA version 13.3 software (Statsoft, Inc., Tulsa, OK, USA). One-way analysis of variance (ANOVA) followed by Tukey’s post hoc test was used to determine the differences between the mean values of multiple groups. The T-student’s test was applied to determine the significant difference between two independent groups. The equality of variances assumption was verified with the Levene’s test. 

## 3. Results

### 3.1. Polyphenol Composition in the Elderberry Fruit Extract 

**Figure 1:**
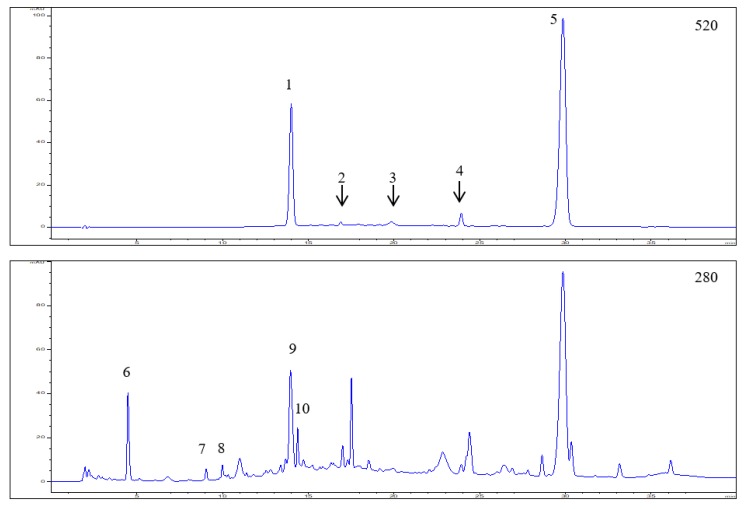

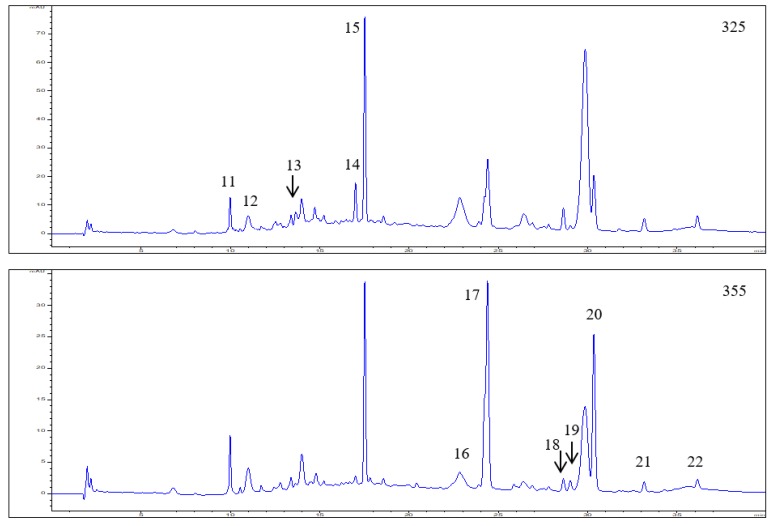
HPLC-DAD chromatograms of elderberry (*Sambucus nigra* L.) fruit extract recorded at 520, 280, 325, and 355 nm, respectively. Peak numbers and retention times refer to compounds indicated in Table 2.

HPLC-DAD-ESI-MS^n^ analysis of the EDB extract revealed the presence of 22 polyphenolic compounds, including anthocyanins (peaks 1–5), hydroxybenzoic acid derivative (peak 6), flavan-3-ols (peaks 7–8), polymers, tentatively identified as hydrolysable tannins (peaks 9–10), hydroxycinnamic acids (peaks 11–15), and flavonols (peaks 16–22). HPLC-DAD chromatograms and chromatographic characteristics with mass spectral data of polyphenols identified in the EDB extract are presented in Figure 1 and Table 2, respectively. 

Anthocyanins accounted for 43% of all polyphenolics; cyanidin-based anthocyanin compounds (-3,5-*O*-diglucoside, -3-*O*-sambubiosyl-5-*O*-glucoside, -3-*O*-glucoside, -3-*O*-rutinoside, and -3-*O*-sambubioside) were the main group of anthocyanins, with a significant predominance of cyanidin-3-*O*-sambubioside ([M + H]^+^ at *m*/*z* 287) constituting 73.2% of all anthocyanins. Peak 1 contained two compounds, identified as cyanidin-3,5-*O*-diglucoside ([M + H]^+^ at *m*/*z* 611) and cyanidin-3-*O*-sambubiosyl-5-*O*-glucoside ([M + H]^+^ at *m*/*z* 743). These anthocyanins represented 25.0% of total anthocyanin compounds, quantitatively determined in the EDB extract. In contrast, cyanidin-3-*O*-glucoside ([M + H]^+^ at *m*/*z* 449) and cyanidin-3-*O*-rutinoside ([M + H]^+^ at *m*/*z* 595) were only detected in trace amounts in the EDB extract (Table 2). The anthocyanin with a molecular ion of *m*/*z* 433, that yielded on MS^2^ fragment at *m*/*z* 271, was identified as pelargonidin-3-*O*-glucoside and quantified in small amounts estimated at 2.3% of all anthocyanins.

Other groups of compounds: Flavan-3-ols, hydroxycinnamic acids, and flavonols amounted to 22.1%, 16.0%, and 13.7% of the total content of polyphenols, respectively. Moreover, the presence of 4-hydroxybenzoic acid glucoside (5.2%) was found in the extract. The group of non-anthocyanin compounds with the largest share in the polyphenol pool were flavan-3-ols, among which catechin and epicatechin (36%), and tannins (64%) were identified. A total of five compounds were detected within another group of hydroxycinnamic acid derivatives, including *p*-coumaric acid hexoside (57%), *p*-coumaroylquinic acid (16%), chlorogenic acid, and its isomers: Neochlorogenic and cryptochlorogenic acids (27%). Concerning flavonols, the results of the HPLC-DAD analysis revealed the presence of quercetin, kaempferol, and isorhamnetin derivatives, with quercetin-3-*O*-rutinoside (53%) and kaempferol-3-*O*-rutinoside (32%) quantified as the dominant compounds within this class. 

The polyphenolic compounds, described above, have been previously identified in berries of *Sambucus nigra* [7,23,24]. However, the content of individual polyphenols in EDB fruits varies, depending on the EDB genotypes as well as specific growth conditions. The total polyphenol content in the extract analyzed was determined to 31.03 mg/g of EDB lyophilized powder, including 13.34 mg of anthocyanins, 6.85 mg of flavan-3-ols, 4.98 mg of hydroxycinnamic acid derivatives, 4.26 mg of flavonols, and 1.6 mg of hydroxybenzoic acid glucoside (Table 2). 

### 3.2. Digestive Enzyme Activity Inhibition by Elderberry Fruit Extract

**Figure 2:**
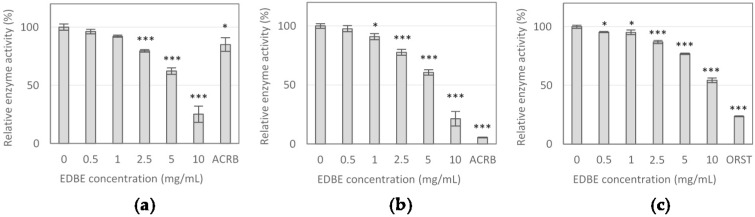
The effect of elderberry fruit extract (EDBE) on the activity of digestive enzymes: α-glucosidase (**a**), α-amylase (**b**), and lipase (**c**). Enzyme activity is expressed in relation to the negative control (without extract addition). As positive controls, reference enzyme inhibitors, including acarbose (ACRB, 100 μg/mL) and orlistat (ORST, 5 μg/mL) were used in the experiment. The values represent the means (*n* = 3) ± SD. * *p* < 0.05, *** *p* < 0.001 vs. control group.

The EDB extract was evaluated for the ability to inhibit digestive enzymes, including α-glucosidase, α-amylase and lipase. The extract showed similar α-glucosidase and α-amylase inhibitions (Figure 2a,b), with the same IC_10_ value of 1.25 mg/mL and non-significantly different IC_50_ values of 6.38 mg/mL and 6.70 mg/mL, respectively (Table 3). 

Lower potency of the extract was observed in inhibiting pancreatic lipase activity. The extract concentrations reducing lipase activity by 10% and 50% were determined at 2.09 mg/mL and 10.98 mg/mL, respectively (Table 3). In inhibiting α-amylase and lipase activity, the drugs acarbose (100 μg/mL) and orlistat (5 μg/mL) were more potent than the EDB extract (Figure 2b,c). However, in α-glucosidase inhibition, the extract at concentrations of 5 mg/mL and 10 mg/mL evoked the stronger effects than acarbose at a dose of 100 μg/mL.

### 3.3. The Effect of Elderberry Fruit Extract on Hypertrophied Adipocytes

**Figure 3:**
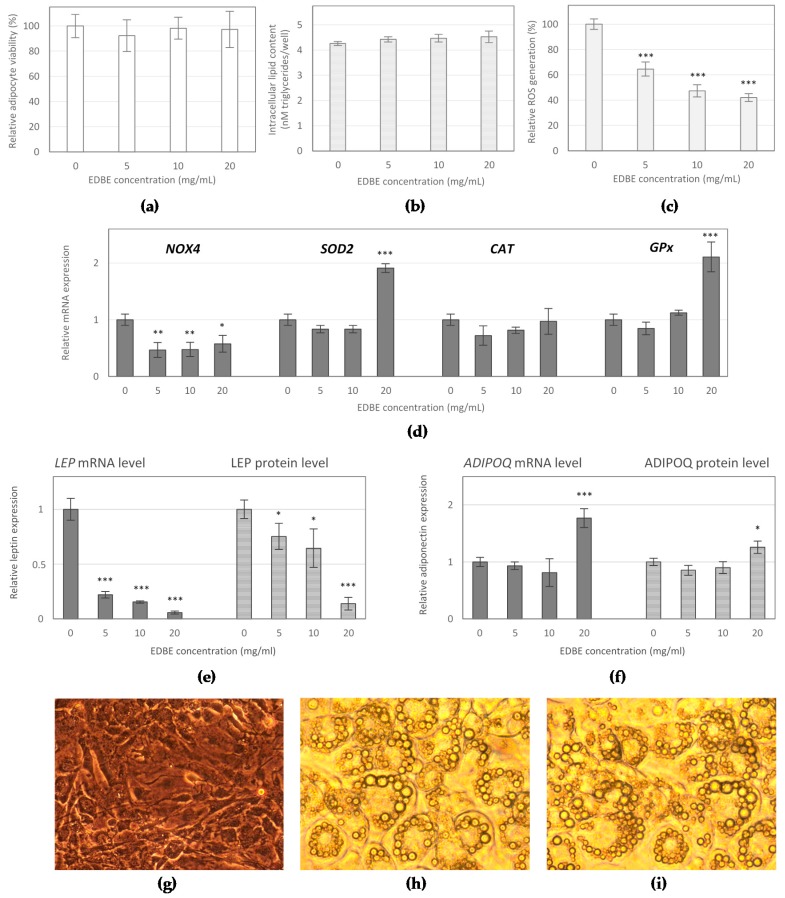
Changes in cell viability (**a**), intracellular triglyceride content (**b**), reactive oxygen species production (**c**), and mRNA expression of *NOX4*, *SOD2*, *catalase* (*CAT*), and *GPx* enzymes (**d**) as well as leptin (*LEP*) (**e**) and adiponectin (*ADIPOQ*) (**f**) expression upon the treatment of3T3-L1 mature adipocytes with the elderberry fruit extract (EDBE). The photos present 3T3-L1 preadipocytes (**g**), fully differentiated 3T3-L1 adipocytes non-treated (**h**) and treated with EDBE at the concentration of 20 mg/mL (**i**). The cells were photographed at magnification of 100 ×. The results were expressed as the means ± SD (*n* = 3). * *p <* 0.05, ** *p <* 0.01, *** *p <* 0.001 vs. control group.

To determine whether EDB extract affects the condition of mature adipocytes, we examined its effect on the viability, lipid accumulation, and ROS production, as well as regulation of leptin and adiponectin expression in terminally differentiated 3T3-L1 cells. The obtained results indicated that EDB extract did not exert any significant effect on both adipocyte viability (Figure 3a) and intracellular lipid content (Figure 3b,g–i). Nevertheless, it dose-dependently inhibited the intracellular ROS generation in hypertrophied adipocytes. EDB extract caused 36%, 53%, and 58% decrease in ROS level when applied at 5, 10, and 20 mg/mL, respectively, in comparison to untreated cells (*p* < 0.001) (Figure 3c). Moreover, treatment with EDB extract led to down-regulation of *NADPH oxidase 4* (*NOX-4*) mRNA expression by approximately 49 ± 6%, independently of the extract dose. Treatment with the extract at the maximum concentration (20 mg/mL) resulted in approximately 2-fold increased mRNA expression of *superoxide dismutase* (*SOD*) and *glutathione peroxidase* (*GPx*). In contrast, the extract did not influence the mRNA expression level of *catalase* (*CAT*) (Figure 3d). 

Supplementation of the fully differentiated adipocyte cultures with EDB extract considerably affected the expression of key adipokines in treated adipocytes. The decrease in *leptin* (*LEP*) mRNA levels between 78% and 94% was observed in mature adipocytes exposed to the extract at concentrations ranging from 5 mg/mL to 20 mg/mL (*p* < 0.001) (Figure 3e). In contrast to leptin, the expression of adiponectin was significantly up-regulated. The highest dose of EDB extract elevated *adiponectin* (*ADIPOQ*) mRNA level by 77% compared to the control (*p* < 0.001) (Figure 3f). The exposure of mature adipocytes to EDB extract resulted in a decrease of leptin secretion. The extract at concentrations of 5 mg/mL and 10 mg/mL significantly reduced leptin synthesis (*p* < 0.05). Whereas, the highest inhibitory effect with the reduction of leptin by 86% (*p* < 0.001) was observed in the cells treated with the extract at maximum concentration. In contrast, EDB extract at the highest dose of 20 mg/mL stimulated the adiponectin secretion in treated cells. The level of adiponectin was increased by 36% respect to the control (*p* < 0.05) (Figure 3f).

### 3.4. The Effect of Elderberry Fruit Extract on Glucose Uptake in Mature 3T3-L1 Adipocytes

**Figure 4:**
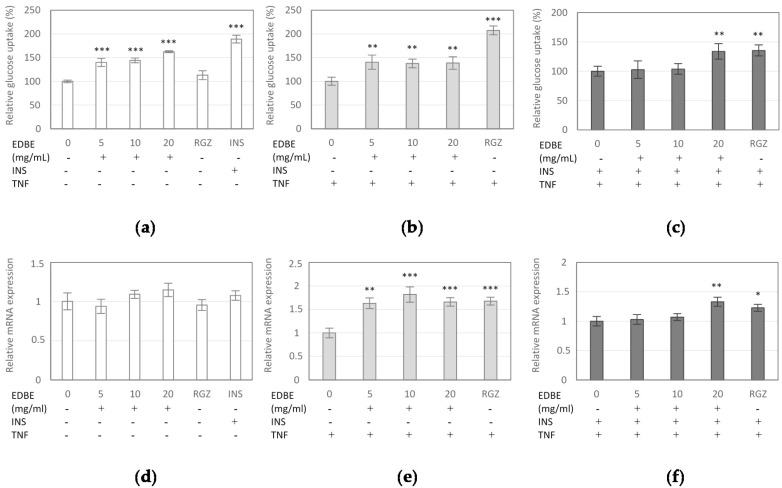
Effect of the elderberry fruit extract (EDBE) on the glucose uptake (**a**–**c**) and glucose transporter *GLUT-4* mRNA expression (**d**–**f**) in mature 3T3-L1 adipocytes non-induced (**a**,**d**) and induced by TNF-α (**b–f**) and non-treated (**b**,**e**) and treated (**c**,**f**) with insulin (INS). The cells were exposed to EDBE at concentrations of 5, 10, and 20 mg/mL, and to rosiglitazone (RGZ). Data are mean values ± SD (*n* = 3). The significance of the main effects of EDBE was determined by Tukey post hoc test; the control (INS, RGZ) significance was analyzed by T-student test; * *p* < 0.05, ** *p* < 0.01, *** *p* < 0.001.

The effect of EDB extract on the glucose analogue (2-NBDG) uptake in mature 3T3-L1 adipocytes was analyzed to determine whether the extract affects the glucose uptake by adipocytes. As shown in Figure 4a, EDB extract caused a significant increase in 2-NBDG uptake at all assayed concentrations (*p* < 0.001). The extract stimulated 2-NBDG uptake by 40%, 44%, and 62% tested at 5, 10, and 20 mg/mL, respectively, in comparison to the control system. Unexpectedly, the stimulatory effect of the extract on the glucose uptake in insulin-sensitive cells was more effective than that observed with rosiglitazone (Figure 4a). Subsequently, the expression of *glucose transporter type 4* (*GLUT-4*) gene in mature 3T3-L1 cells exposed to EDB extract was investigated. The results indicated no significant effect of EDB extract on *GLUT-4* mRNA level (Figure 4d).

In the parallel experiment, the influence of EDB extract on 2-NBDG incorporation into insulin resistant adipocytes treated with TNF-α was determined. The cells were incubated with the extract in the absence or presence of insulin at 100 nM. In the insulin-resistant adipocytes cultured without insulin supplementation, the EDB extract at concentrations of 5, 10, and 20 mg/mL enhanced 2-NBDG uptake by 40%, 38%, and 39%, respectively (Figure 4b). In this case, quantitative PCR analysis revealed that treatment with EDB extract at doses from 5 mg/mL to 20 mg/mL significantly up-regulated the mRNA expression of *GLUT-4* with an increase ranging from 63% to 82% (Figure 4e). 

In the experiments on insulin resistant adipocytes exposed to insulin, the EDB extract at the highest concentration showed insulin-sensitizing properties, stimulating 2-NBDG incorporation by 34% (*p* < 0.01). Its efficacy was comparable to that of rosiglitazone, which enhanced the 2-NBDG uptake by 36% (*p* < 0.01) (Figure 4c). In this study, the extract at a dose of 20 mg/mL up-regulated the expression of *GLUT-4* by 33% (*p* < 0.01), compared to control adipocytes (Figure 4f).

### 3.5. Anti-Inflammatory Effects of Elderberry Fruit Extract

**Figure 5:**
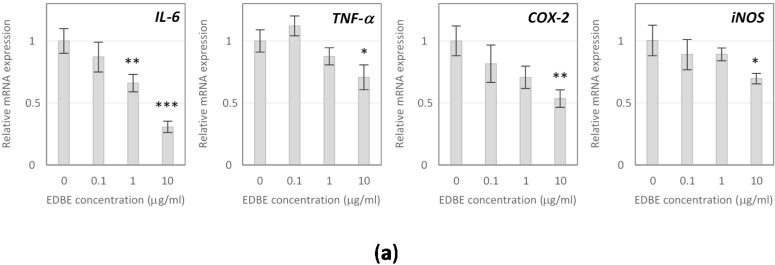

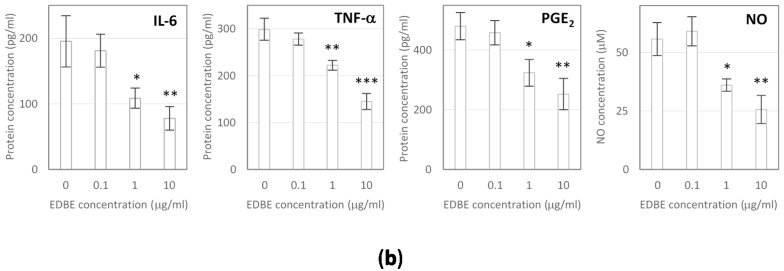
Effect of the elderberry fruit extract (EDBE) on mRNA expression of *IL-6*, *TNF-α*, *COX-2,* and *iNOS* (**a**) and on the production of IL-6, TNF-α, PGE_2_ protein, and NO (**b**) in the lipopolysaccharide (LPS)-activated RAW 264.7 macrophages. Data are mean values ± SD (*n* = 3). * *p* < 0.05, ** *p* < 0.01, *** *p* < 0.001 when compared with control.

Anti-inflammatory effects of EDB extract were evaluated in activated RAW 264.7 macrophages, considering proinflammatory cytokines and mediators determined at both molecular and cellular levels, using low non-cytotoxic extract doses of 0.1, 1, and 10 µg/mL. The obtained results demonstrated the ability of EDB extract to alleviate the cellular inflammatory response induced by LPS. The extract at concentrations of 1 and 10 µg/mL suppressed mRNA expression of *IL-6* by 34% (*p* < 0.01) and 69% (*p* < 0.001), respectively, compared to control macrophages. Moreover, a 28% decrease in *TNF-α* mRNA level (*p* < 0.05) was observed following treatment with EDB extract at a dose of 10 µg/mL (Figure 5a). The down-regulation of *IL-6* and *TNF-α* expression was consistent with the inhibited secretion of these cytokines. Namely, EDB extract assayed at 1 µg/mL reduced production of IL-6 and TNF-α by 44% and 26%, respectively (*p* < 0.05). At the extract concentration of 10 µg/mL, the 60% decrease in IL-6 secretion (*p* < 0.01) was observed while synthesis of TNF-α was reduced by 52% (*p* < 0.001) (Figure 5b). 

The extract at a dose of 10 µg/mL also affected *COX-2* expression, causing a 46% reduction of *COX-2* transcripts level in activated macrophages (*p* < 0.01) (Figure 5a). As a consequence, the decrease in PGE_2_ production was detected after cell exposure to the extract. Namely, PGE_2_ level lowered by 33% and 47%, respectively, for a dose of 1 µg/mL and 10 µg/mL (Figure 5b).

Furthermore, the obtained results demonstrated the reduction in NO production as well as down-regulation of *inducible NO synthase* (*iNOS*) expression in LPS-stimulated RAW 264.7 macrophages in response to the EDB extract treatment. The extract reduced the NO synthesis by 35% (*p* < 0.05) when assayed at 1 µg/mL and by 54% (*p* < 0.01) when applied at 10 µg/mL (Figure 5b). A significant inhibitory effect of EDB extract on *iNOS* expression was noted only at the highest tested dose, which decreased *iNOS* mRNA level by 30% (*p* < 0.01) (Figure 5a). 

## 4. Discussion

Excessive fat accumulation in hypertrophic adipose tissue associated with obesity is responsible for oxidative stress, chronic inflammation, and dysregulated adipokine secretion [25]. It is believed that the therapeutic potential of natural dietary compounds against obesity and obesity-related disorders should focus on improving the fat function in pathogenic hypertrophic adipocytes by reducing oxidative stress, alleviating inflammation, and regulating underproduction or overproduction of clinically relevant adipocyte factors. However, most bioactive compounds or extracts strongly affect preadipocytes, their viability, proliferation, and differentiation into mature fat cells, without any significant effects on the pathological status of hypertrophic adipocytes. Therefore, in this work, the influence of the *Sambucus nigra* fruit extract on mature fully differentiated insulin-resistant 3T3-L1 adipocytes was investigated.

In our study, we found no reduction in cell viability and lipid content in hypertrophic 3T3-L1 adipocytes after exposure to EDB extract. However, as a result of the treatment, the intracellular ROS generation was significantly down-regulated and probably, oxidative stress accompanying excessive fat accumulation was also importantly reduced. Oxidative stress induced by enhanced lipid content is reported to be involved in the pathogenesis of obesity-related comorbidities including insulin resistance and diabetes, cardiovascular complications, and cancer [26]. It was found that ROS are intensively generated in visceral adipose tissue by adipocytes during the metabolism of excess nutrients and also by macrophages, which accumulate in adipose tissue in obesity state. The increased release of fatty acids from overproduced fat accumulated in adipose tissue, activate NADPH oxidases (NOX) and induce or aggravate ROS production. Other factors that also contribute oxidative stress to obesity include hyperleptinemia, low antioxidant defense, or chronic inflammation [27]. Our results showed that EDB extract could reduce ROS generation by lowering the expression of NOX4, the major NOX isoform in adipocytes. Treatment of hypertrophied 3T3-L1 adipocytes with EDB extract caused a significant decreasing in *NOX4* mRNA expression. Furthermore, up-regulation of mRNA expression of antioxidant enzymes, like SOD and GPx, could also contribute to enhancing adipocyte antioxidant defense efficiency. Numerous studies have shown the high antioxidant capacity of *Sambucus nigra* fruit [6,10]. However, the antioxidant effects of EDB on adipocytes have not yet been reported in the literature. In the present study, we demonstrated that introduction of EDB extract to the culture of hypertrophic adipocytes resulted in decreased ROS generation in cells. The antioxidant action of EDB extract in adipocytes may be a potential protective mechanism against obesity-associated pathological risk factors, including insulin resistance and chronic inflammation.

Additionally, EDB extract treatment modulated the leptin and adiponectin gene expression and protein secretion in hypertrophic 3T3-L1 adipocytes. Leptin and adiponectin are adipocytokines, which influence energy homeostasis, glucose and lipid metabolism, cardiovascular function, and immune response [28]. Leptin is primarily secreted by fully differentiated adipocytes, and its crucial role is to regulate energy intake and expenditure through controlling appetite and glucose metabolism. Reflecting the increased amount of adipose tissue, obese individuals often have elevated leptin concentration and the simultaneous apparent loss of efficacy of leptin, which is a result of leptin resistance, the state that leads to uncontrolled food intake, pro-inflammatory state, diabetes mellitus, and other obesity-related complications [29]. In contrast to leptin, adiponectin is down-regulated in obesity, and the circulating adiponectin levels are inversely correlated with body fat amount. Adiponectin enhances energy metabolism and fatty acid oxidation, promotes insulin sensitivity, improves glucose tolerance, and exerts anti-inflammatory effects [28]. Low serum adiponectin and high serum leptin levels are considered as risk factors for developing type 2 diabetes (T2DM), obesity, dyslipidemia, hypertension, and cardiovascular diseases. In this study, a remarkable decrease in leptin expression and secretion was observed in response to EDB extract treatment of hypertrophied 3T3-L1 adipocytes, which may help counteract the leptin resistance state. Whereas, adiponectin mRNA expression and protein secretion in treated adipocytes were significantly increased. The effect of EDB extract on adiponectin production may indicate anti-inflammatory potential and insulin-sensitizing activity of *Sambucus nigra* fruit. 

The association of visceral obesity with T2DM is a long-recognized phenomenon. The primary determinant of this correlation is the fact that central obesity is the critical factor in the emergence of insulin resistance. The insulin-resistant state results in defective insulin-stimulated glucose uptake and consequently in hyperglycemia, elevated circulating free fatty acids level, abnormal fat accumulation, and dysregulation of hepatic glucose production, that, in combination with a paucity of insulin secretion by pancreatic β-cells, leads to T2DM [30]. These metabolic abnormalities may arise from impairment in insulin signaling pathways and subsequent defect in translocation of insulin-responsive glucose transporter protein (GLUT-4) and in adipose tissue, also from down-regulation of *GLUT-4* gene [31].

The effects of EDB extract on glucose uptake and *GLUT-4* expression were evaluated in this study. Experiments were performed both with mature 3T3-L1 adipocytes sensitive to insulin and adipocytes treated with TNF-α to induce an inflammatory status and insulin resistance. Analysis revealed that EDB extract stimulated the 2-NBDG uptake in both types of adipocytes and up-regulated mRNA expression of *GLUT-4* in insulin-resistant cells, suggesting insulin-like and insulin-sensitizing activities of the extract. The signaling pathways involved in the development of these activities will be further examined in future studies. This is the first study assessing the effects of EDB extract on glucose uptake in 3T3-L1 cells. Although several recent reports have suggested the anti-diabetic and hypoglycemic properties of elderberry, it has been found that EDB methanolic extracts markedly stimulate glucose uptake in liver HepG_2_ cells and also exert inhibitory effect towards carbohydrate hydrolyzing enzyme [32]. Furthermore, EDB extracts, EDB anthocyanins, mainly cyanidin-3-glucoside and cyanidin-3-sambubioside, procyanidins, and their metabolites were found to enhance glucose uptake in human skeletal muscle cells [33]. Whereas, EDB lipophilic and polar extracts were reported to modulate glucose metabolism or lower insulin secretion contributing to the mitigation of insulin resistance in T2DM rats [13]. 

Anti-obesity and anti-diabetic activity of EDB extract could be related to the inhibition of dietary fat and sugar absorption from the intestinal tract. There is some evidence that polyphenols from berry fruits, such as strawberry, raspberry, blueberry, bilberry, black and red currant, lingonberry, red and green gooseberry, cranberry, and chokeberry, contribute to the inhibition of digestive enzymes involved in the hydrolysis of dietary lipids and carbohydrates [34]. Based on our research, the *Sambucus nigra* fruit may be included in the class of berries considered as effective inhibitors of α-amylase, α-glucosidase, and pancreatic lipase activity. 

Obesity is known to be accompanied by metaflammation—low-grade chronic inflammation condition triggered by excess nutrients in metabolic cells [35]. An attribute of obesity-related inflammation is enhanced infiltration of macrophages into expanding adipose tissue, activation of specialized immune cells, and secretion of proinflammatory cytokines such as TNF-α, IL-6, and MCP-1 leading to an unresolved inflammatory response, which affects normal metabolism and insulin action [35]. Inhibition of obesity-induced inflammation could, thus, be a therapeutic intervention against adipose tissue dysfunction and related co-morbidities. In recent years, the use of anti-inflammatory nutrients provided through diet as a potential approach against obesity has been extensively studied [36,37]. 

In the present study, we evaluated anti-inflammatory effects of EDB extract in LPS-stimulated RAW 264.7 macrophages. Activated macrophages produce cytokines such as TNF-α, IL-1β, and IL-6 as well as pro-inflammatory mediators, such as NO and PGE_2_ [38]. IL-6 and TNF-α are potent proinflammatory cytokines, which play a central role in inflammatory response and are characterized by a broad spectrum of functions with various effects in adipose tissue. TNF-α substantially influences lipid metabolism and adipocytes apoptosis. It can disrupt insulin signaling pathway promoting insulin resistance and adipocytes dysfunction [39]. TNF-α has, thus, been believed to be the crucial mediator in the detrimental paracrine loop between adipocytes and macrophages [40]. IL-6 has a pivotal role in acute phase reactions. It also influences hormonal balance and energy homeostasis and may affect the increase of free fatty acids level. Circulating levels of IL-6 and TNF-α are elevated in obese individuals and patients with insulin resistance [41]. In general, the regulation of TNF-α and IL-6 secretion is considered to be a potent treatment strategy for inflammation-associated diseases [42]. 

The research presented in this work suggests that EDB extract dose-dependently down-regulates mRNA expression and protein production of TNF-α and IL-6 in activated RAW 264.7 macrophages and therefore alleviates the cellular inflammatory response induced by LPS. In addition to TNF-α and IL-6, EDB extract significantly reduced the production of inflammatory mediators—PGE_2_ and NO. Increased level of PGE_2_ is observed in obese adipose tissue due to remarkable up-regulation of COX-2—the key enzyme in eicosanoid metabolism, of which expression is induced in inflammation state [43]. It has been suggested that COX-2-mediated inflammation in visceral fat is responsible for insulin resistance and fatty liver development in high-fat-induced obese rats [44]. The same study revealed that COX-2 inhibition significantly reversed adipocyte hypertrophy, macrophage infiltration, and decreased markers of adipocyte differentiation. Nitric oxide formed by iNOS is a short-lived vasodilator that acts as an important regulator of physical homeostasis, while its overproduction has been closely correlated with the pathological conditions including septic shock, osteoporosis and rheumatoid arthritis, insulin resistance, and inflammation [45]. In the present study, EDB extract was found to suppress PGE_2_ and NO production via down-regulation of *COX-2* and *iNOS* expression. These findings indicate that inhibition of PGE_2_ and NO generation is one of the anti-inflammatory mechanisms of the extract. Several recent studies have shown the anti-inflammatory potential of EDB fruit preparations. In our previous study, we demonstrated the anti-inflammatory potential of gastrointestinally digested EDB extract following intestinal absorption in a co-culture model of intestinal epithelial Caco-2 cells and LPS-stimulated RAW 264.7 macrophages [7]. The analyzed extract down-regulated the expression of genes (IL-1β, IL-6, TNF-α, COX-2) involving in the inflammatory pathway in a range comparable to that of budesonide. This study demonstrated adequate bioavailability and intestinal permeability of EDB compounds that are probably sufficient to evoke systemic anti-inflammatory effects [7]. Moreover, there is increasing evidence that the EDB bioactives can penetrate the blood–brain barrier and modulate the immune response induced in different types of brain injuries, including ischemic stroke. It has been found that EDB extract and its phenolic components significantly inhibit activation of microglia, considered to be resident macrophages responsible for the initial immune response to brain injuries. Treatment of activated microglial bv-2 cells with EBD extract led to diminishing ROS and NO generation, and as a consequence, attenuating the neuroinflammatory process [16].

Results of the study, as discussed above, indicate that *Sambucus nigra* fruit extract may offer substantial preventive and therapeutic potential for the treatment of obesity and obesity-related disorders, accompanied by oxidative stress, inflammationm and insulin resistance. Moreover, the extract can inhibit digestive enzyme activity, and consequently, significantly reduce the intestinal absorption of dietary lipids and carbohydrates, which is an effective strategy for the prevention and treatment of obesity and metabolic comorbidities.

Considering the findings of *in vitro* studies, we can postulate a nutraceutical application of the *Sambucus nigra* fruit extract. The scientific community focuses great attention on introducing nutraceuticals into the daily diet to prevent the occurrence of the pathological conditions, to delay or avoid the need for drug treatment and to support pharmacological therapy. Nutraceuticals as pharmafoods should be evaluated in the clinical aspects regarding safety, side effects, bioavailability, beneficial health effects, mechanisms of action and efficacy, and any possible interactions between food and drugs assumed together with them [46,47]. Thus, the developing of clinical studies will be of significant importance for clinically justified promotion of the *Sambucus nigra* fruit extract as a safe nutraceutical with the capacity of prevention or treatment of obesity and obesity-related immune-metabolic disorders.

## Figures and Tables

**Table 1:** The primers sequence used for real-time PCR.

Gene	Accession	No. Sequence (5′–3′)	Amplicon (bp)
Mm *LEP*	NM-008493	F: GGA TCA GGT TTT GTG GTG CT	187
		R: TTG TGG CCC ATA AAG TCC TC	
Mm *GLUT-4*	NM-001359114.1	F: TGC TGG GCA CAG CTA CCC	162
		R: CGG TCA GGC GCT TTA GAC	
Mm *ADIPOQ*	NM-009605	F: CTG GCC ACT TTC TCC TCA TT TC	120
		R: GGC ATG ACT GGG CAG GAT TA	
Mm *IL-6*	NM-031168.1	F: TCT GAA GGA CTC TGG CTT TG	142
		R: GAT GGA TGC TAC CAA ACT GGA	
Mm *NOS-2*	NM-010927.3	F: TGA AGA AAA CCC CTT GTG CT	100
		R: TTC TGT GCT GTC CCA GTG AG	
Mm *PTGS2*	NM-011198.3	F: GGC GCA GTT TAT GTT GTC TGT	107
		R: CAA GAC AGA TCA TAA GCG AGG A	
Mm *TNF-α*	NM-001278601.1	F: AGG GTC TGG GCC ATA GAA CT	103
		R: CCA CCA CGC TCT TCT GTC TAC	
Mm *NOX-4*	NM-015760.5	F: GAT CAC AGA AGG TCC CTA GCA G	134
		R: GTT GAG GGC ATT CAC CAA GT	
Mm *SOD2*	NM-013671.3	F: CGT GTC TGT GGG AGT CCA AGG TTC AG	139
		R: GTC AAT CCC CAG CAG CGG AAT AAG	
Mm *CATALASE*	NM-009804.2	F: CCT CCT CGT TCA GGA TGT GGT T	243
		R: CGA GGG TCA CGA ACT GTG TCA G	
Mm *GPx*	NM-008160.6	F: GGG CAA GGT GCT GCT CAT TG	269
		R: AGA GCG GGT GAG CCT TCT CA	
Mm *ACTB*	NM-007393	F: CCA CAG CTG AGA GGG AAA TC	193
		R: AAG GAA GGC TGG AAA AGA GC	

**Table 2:** HPLC-MS identification of phenolic compounds in elderberry fruit extract in positive (electrospray ionization (ESI) +) and negative (ESI −) ionization mode.

Peak No.	RT (min)	UV λ _max_ (nm)	[M]^+^/[M + H]^+^ (*m*/*z*)	[M − H]^−^ (*m*/*z*)	MS/MS (*m*/*z*)	Tentative Identification	Concentration (mg/g) *
1	14.01	280, 520	611.1651	-	287.0583	Cyanidin-3,5-*O*-diglucoside	3.27 ± 0.25
			743.2095		287.0579	Cyanidin-3-*O*-sambubiosyl-5-*O*-glucoside (co-elution)	
2	16.90	280,520	449.1133		287.0632	Cyanidin-3-*O*-glucoside	Trace amounts
3	19.84	280,520	595.1734		287.0578	Cyanidin-3-*O*-rutinoside	Trace amounts
4	23.92	280,520	433.1187		271.0640	Pelargonidin-3-*O*-glucoside	0.31 ± 0.04
5	29.85	280,520	581.1635		287.0633	Cyanidin-3-*O*-sambubioside	9.76 ± 0.68
6	4.48	275		299.2506	—	4-Hydroxybenzoic acid glucoside	1.60 ± 0.12
7	9.05	280		289.1139	245.1203	(+)/(−)-Catechin	1.07 ± 0.06
8	9.99	280		289.1140	245.1210	(+)/(−)-Epicatechin	1.41 ± 0.08
9	13.96	268		597.4616	—	Hydrolysable tannin	3.45 ± 0.22
10	14.38	268		597.4628	—	Hydrolysable tannin	0.92 ± 0.06
11	9.99	299,325		353.2873	191.1737	Neochlorogenic acid	0.50 ± 0.03
12	11.00	299,325		353.2886	191.1749	Chlorogenic acid	0.59 ± 0.04
13	12.54	300,325		353.2865	191.1729	Cryptochlorogenic acid	0.26 ± 0.02
14	17.00	310,234		337.0917	173.0443	*P*-coumaroylquinic acid	0.78 ± 0.06
15	17.52	316,234		371.3016	163.0396	*P*-Coumaric acid hexoside	2.85 ± 0.18
16	22.83	268,354		463.0882	301.0354	Quercetin-3-*O*-glucoside	0.29 ± 0.01
17	24.39	255,355		609.1461	301.0349	Quercetin-3-*O*-rutinoside	2.27 ± 0.19
18	28.63	255,358		505.0872	301.0366	Quercetin 3-*O*-(6”-acetyl-glucoside)	0.09 ± 0.01
19	29.02	266,348		447.0935	285.0540	Kaempferol-3-*O*-glucoside	0.09 ± 0.01
20	30.34	255,352		593.1515	285.0542	Kaempferol-3-*O*-rutinoside	1.36 ± 0.12
21	33.15	255,370		301.0356	151.0031	Quercetin	0.09 ± 0.02
22	36.14	255,352		623.1044	315.0449	Isorhamnetin-3-*O*-rutinoside	0.07 ± 0.01

* mg/g of lyophilized elderberry powder, values were expressed as mean ± SEM for three independent experiments.

**Table 3:** Inhibitory concentrations IC_10_ and IC_50_ (the inhibitor concentration required for 10% and 50% inhibition of enzyme activity) of the elderberry fruit extract (EDBE), acarbose (ACRB), and orlistat (ORST) as reference pharmacological inhibitors.

Enzyme Inhibitor	α-Glucosidase		α-Amylase		Lipase	
	IC_10_	IC_50_	IC_10_	IC_50_	IC_10_	IC_50_
EDBE (mg/mL)	1.25 ± 0.09	6.70 ± 0.56	1.25 ± 0.10	6.38 ± 0.44	2.09 ± 0.30	10.98 ± 0.47
ACRB (μg/mL)	85.12 ± 0.63	> 100	1.30 ± 0.06	8.23 ± 0.16	-	-
ORST (μg/mL)	-	-	-	-	0.2 ± 0.02	1.43 ± 0.15
